# Astrocyte matricellular proteins that control excitatory synaptogenesis are regulated by inflammatory cytokines and correlate with paralysis severity during experimental autoimmune encephalomyelitis

**DOI:** 10.3389/fnins.2015.00344

**Published:** 2015-10-09

**Authors:** Pennelope K. Blakely, Shabbir Hussain, Lindsey E. Carlin, David N. Irani

**Affiliations:** Holtom-Garrett Program in Neuroimmunology, Department of Neurology, University of Michigan Medical SchoolAnn Arbor, MI, USA

**Keywords:** SPARCL1, SPARC, EAE, synaptic plasticity, astrocytes, multiple sclerosis

## Abstract

The matricellular proteins, secreted protein acidic and rich in cysteine (SPARC) and SPARC-like 1 (SPARCL1), are produced by astrocytes and control excitatory synaptogenesis in the central nervous system. While SPARCL1 directly promotes excitatory synapse formation *in vitro* and in the developing nervous system *in vivo*, SPARC specifically antagonizes the synaptogenic actions of SPARCL1. We hypothesized these proteins also help maintain existing excitatory synapses in adult hosts, and that local inflammation in the spinal cord alters their production in a way that dynamically modulates motor synapses and impacts the severity of paralysis during experimental autoimmune encephalomyelitis (EAE) in mice. Using a spontaneously remitting EAE model, paralysis severity correlated inversely with both expression of synaptic proteins and the number of synapses in direct contact with the perikarya of motor neurons in spinal gray matter. In both remitting and non-remitting EAE models, paralysis severity also correlated inversely with *sparcl1:sparc* transcript and SPARCL1:SPARC protein ratios directly in lumbar spinal cord tissue. *In vitro*, astrocyte production of both SPARCL1 and SPARC was regulated by T cell-derived cytokines, causing dynamic modulation of the SPARCL1:SPARC expression ratio. Taken together, these data support a model whereby proinflammatory cytokines inhibit SPARCL1 and/or augment SPARC expression by astrocytes in spinal gray matter that, in turn, cause either transient or sustained synaptic retraction from lumbar spinal motor neurons thereby regulating hind limb paralysis during EAE. Ongoing studies seek ways to alter this SPARCL1:SPARC expression ratio in favor of synapse reformation/maintenance and thus help to modulate neurologic deficits during times of inflammation. This could identify new astrocyte-targeted therapies for diseases such as multiple sclerosis.

## Introduction

Multiple sclerosis (MS) is a common autoimmune disorder of the central nervous system (CNS), and most patients come to medical attention with a relapsing-remitting form of disease (RRMS). Pathological examination of affected CNS tissue shows focal areas of demyelination, often associated with underlying axonal damage, accompanied by varying amounts of immune cell infiltration. The formation of new demyelinating lesions is widely presumed to be the main tissue substrate of clinical relapses, and magnetic resonance imaging (MRI) scans of the brain and spinal cord can detect even small demyelinating lesions in RRMS patients in real time. MRI scans obtained following the intravenous infusion of a paramagnetic contrast material also reveal active blood-brain barrier (BBB) breakdown in white matter plaques, and clinical-radiological-pathological correlations show that these contrast-enhancing lesions represent new areas of active immune-mediated demyelination (Li et al., 2006; Filippi et al., 2012; Sormani and Bruzzi, 2013; Simon, 2014). Clinical remission is associated with a resolution of BBB breakdown on MRI scans (Li et al., 2006; Filippi et al., 2012; Sormani and Bruzzi, 2013; Simon, 2014), but the cellular and molecular substrates of recovery following an MS exacerbation still remain poorly understood. In the experimental autoimmune encephalomyelitis (EAE) model of MS, a relapsing-remitting disease can be provoked in SJL mice following immunization with a peptide of proteolipid protein (PLP) or via the adoptive transfer of polarized CD4+ T cells derived from PLP-immunized mice. This experimental system has been extensively studied to gain mechanistic insight into MS relapses and remissions.

Based on growing evidence of gray matter pathology in MS (Vercellino, 2005; Stadelmann et al., 2008; Geurts et al., 2012; Calabrese et al., 2015), the occurrence and persistence of synaptic abnormalities has become an emerging area of investigation in both MS and EAE. Numerous studies now show direct tissue evidence of reduced synapse density (Vercellino, 2005; Stadelmann et al., 2008; Michailidou et al., 2015), or reduced expression of pre- and post-synaptic proteins (Vercellino, 2005), in gray matter lesions from MS autopsy specimens. Such investigations are, however, focused on more advanced stages of disease and by definition cannot assess synaptic function or identify synaptic plasticity over time. In both rat and mouse EAE models, synaptic abnormalities have been described in the cortex, hippocampus and lumbar spinal cord immediately prior to or just at the time clinical symptoms emerge (Zhu et al., 2003; Marques et al., 2006; Centonze et al., 2009; Freria et al., 2010; Ziehn et al., 2010; Yang et al., 2013; Di Filippo et al., 2015; Mandolesi et al., 2015), and data suggest these synaptic changes can reverse themselves in animals that undergo clinical recovery (Zhu et al., 2003; Marques et al., 2006; Centonze et al., 2009; Freria et al., 2010; Yang et al., 2013; Mandolesi et al., 2015). *In vivo* two-photon microscopy has been used to image axonal boutons and dendritic spines in the cortices of mice with EAE in real time and shows notable synaptic instability driven by the release of soluble inflammatory mediators (Yang et al., 2013). If synaptic and/or dendritic changes are important contributors to the neurological deficits that occur in EAE and MS, then an improved understanding of the molecular mechanisms controlling these events could have therapeutic relevance.

Astrocytes are now recognized to play important roles in synaptic development and plasticity (Clarke and Barres, 2013; Haydon and Nedergaard, 2014; De Pittà et al., 2015). Not only do these cells release transmitters that modulate synaptic activity (Allen and Barres, 2005), but they also control the actual formation, maturation, and elimination of synapses through both secreted and cell contact-dependent mechanisms (Clarke and Barres, 2013). The matricellular proteins, secreted protein acidic and rich in cysteine (SPARC, also known as osteonectin) and SPARC-like 1 (SPARCL1, also known as synaptic cleft 1 (SC1) or Hevin), are produced by astrocytes and regulate excitatory synaptogenesis *in vitro* and in the developing CNS *in vivo* (Kucukdereli et al., 2011). While SPARCL1 directly promotes excitatory synapse formation, SPARC specifically antagonizes the synaptogenic activity of SPARCL1 (Kucukdereli et al., 2011). Both proteins are normally expressed in the adult CNS (Kucukdereli et al., 2011; Lloyd-Burton and Roskams, 2012), and longitudinal changes in SPARCL1 expression have been documented in the setting of CNS disease; tissue levels decline following experimental seizures but rise after focal ischemia (Lively and Brown, 2008; Lively et al., 2011). Correlation with both the number and function of existing excitatory synapses in these disease settings has not been well reported.

We hypothesized that local tissue inflammation influences synaptic plasticity in ventral gray matter of the lumbar spinal cords of mice with EAE by dynamically regulating astrocyte production of SPARCL1 and SPARC. Changes to the synapses in direct contact with lumbar ventral motor neurons could contribute to the expected hind limb paralysis that is characteristic of this disease. We also speculated that similar immunoregulatory effects could be reproduced *in vitro* using primary astrocyte cultures. Data reported here shed further light on the cellular and molecular underpinnings of reversible neurologic deficits in CNS autoimmune demyelinating diseases.

## Materials and methods

### Animals

Female C57BL/6 and SJL mice (8–10 weeks old) were obtained from Harlan Laboratories (Indianapolis, IN) or the National Cancer Institute (Frederick, MD). Animals were maintained under specific pathogen-free conditions in accordance with guidelines set by the National Institutes of Health (NIH). The University of Michigan Committee on the Use and Care of Animals approved all of our experimental procedures. Mice were housed on a 10 h light/14 h dark cycle in ventilated cages containing up to five animals each. Food and water were available *ad libitum*.

### Animal manipulations

Active EAE was induced in SJL mice by subcutaneous (s.c.) immunization with 100 μg PLP_139−155_ peptide (Biosynthesis, Lewisville, TX) emulsified in complete Freund's adjuvant (CFA, Difco, Detroit, MI) containing an additional 4 mg/ml heat killed *Mycobacterium tuberculosis*, H37Ra (Difco). Active EAE was induced in C57BL/6 mice by s.c. immunization with 100 μg myelin oligodendrocyte glycoprotein (MOG)_35−55_ peptide (Biosynthesis) emulsified in CFA supplemented with 4 mg/ml heat killed *Mycobacterium tuberculosis*, H37Ra. Each C57BL/6 mouse also received 300 ng of pertussis toxin (List Biological Laboratories, Campbell, CA) via intraperitoneal (i.p.) injection on the day of immunization and again 48 h later. Following immunization, animals were observed daily for clinical signs of EAE and were scored by blinded examiners using the following scale: 0 = no signs of disease; 1 = flaccid tail or hind limb weakness; 2 = flaccid tail, hind limb weakness and loss of righting reflex; 3 = partial hind limb paralysis; 4 = complete hind limb paralysis; 5 = moribund or dead.

At predetermined disease stages, some mice were euthanized via extensive transcardiac perfusion with phosphate-buffered saline (PBS) under anesthesia. For Western blots, PCR quantification of RNA transcripts, and ELISA and Luminex assays done on tissue extracts, spinal cords were quickly dissected for further processing. For immunohistochemical and immunofluorescence studies, animals were perfused a second time with chilled PBS containing 4% paraformaldehyde (PFA) before dissection. For electron microscopy (EM) preparations, animals were perfused with 0.1 M Sorenson's buffer, followed by 2.5% glutaraldehyde in Sorenson's buffer before dissection.

### Western blot analyses

Spinal cords were mechanically homogenized in a tissue lysis buffer (10 mM Tris, 1% sodium dodecyl sulfate (SDS), 1 mM sodium orthovandate, pH 7.6) supplemented with a commercial protease inhibitor cocktail (Roche Life Science, Indianapolis, IN). Tissue lysates were centrifuged to remove undigested tissue debris and the total protein concentration of each supernatant determined using the Pierce Coomassie Protein Assay Reagent (Thermo Fisher Scientific, Rockford, IL). Samples were boiled in 4x protein sample buffer and 20 μg/well run on SDS-polyacrylamide gels. Proteins were transferred to PVDF membranes and blocked overnight at 4°C in a 5% non-fat skim milk solution in Tris-buffered saline (TBS) containing 0.5% Tween 20. Membranes were then incubated with a primary antibody (anti-glial fibrillary acidic protein (GFAP), clone GA5 (1:1000, EMD Millipore, Temecula, CA); anti-synaptophysin, clone SP15 (1:500, EMD Millipore); or anti-microtubule associated protein 2 (MAP2), clone AP20 (1:2000, EMD Millipore)) for 1 h at room temperature. Following five sequential washes, membranes were then incubated with a species-specific horseradish peroxidase (HRP)-conjugated secondary antibody (1:10,000, EMD Millipore) for 1 h at room temperature. Membranes were washed again, and the HRP signal detected using ECL Western blotting detection reagent (GE Healthcare Bio-Sciences, Pittsburg, PA). After the chemiluminescent signal of each blot was collected on x-ray film, membranes were stripped using Western blot stripping buffer (Thermo Fisher Scientific) and relabeled with β-actin loading control antibody (1:5000, Thermo Fisher Scientific) using the same steps described above. Once all the β-actin signals were obtained, all band densities were quantified using the ImageJ software package (NIH, Bethesda, MD). The band density for each protein was first normalized to the β-actin signal detected in the same lane, and naïve samples were then set to an arbitrary expression level of 1.0. Relative protein expression across the full course of EAE was determined compared to naïve controls, and expression in 5 samples at each disease stage was analyzed for statistical significance.

### Tissue processing, staining and imaging

Spinal cords were post-fixed for 6 h in 4% PFA in PBS, cryopreserved overnight in 30% sucrose in PBS, and snap frozen in Tissue-Tek CRYO-OCT Compound (Thermo Fisher Scientific). Eight micron frozen sections were cut, collected on SuperFrost Plus slides (Thermo Fisher Scientific) and stored at −20°C until staining. At that time, sections were brought to room temperature, washed in PBS and boiled for 20 min in 0.01 M citric acid in PBS (pH 6.0) to unmask tissue antigens. Tissue sections were then permeabilized for 5 min in 0.1% Triton X-100 in PBS and blocked for 30 min in 5% normal rabbit serum (NRS).

For immunofluorescence, sections were incubated for 1 h at room temperature with goat anti-mouse SPARCL1 antibody (1:250; R&D Systems, Minneapolis, MN). Sections were washed 3 times in PBS and incubated with rhodamine-conjugated rabbit anti-goat secondary antibody (1:200, eBioscience, San Diego, CA) for 1 h at room temperature. Sections were washed again, blocked in 5% normal goat serum (NGS) in PBS, and incubated for 1 h with anti-GFAP monoclonal antibody (1:250, EMD Millipore). After washing 3 times in PBS, slides were incubated with FITC-conjugated goat anti-mouse secondary antibody (1:200, eBioscience). The sections were then washed, nuclei counterstained with DAPI (0.5 μg/ml), coverslipped and imaged using a Nikon Ti-U inverted microscope equipped with a CoolSNAP EZ CCD digital camera (Photometrics, Tucson, AZ) supported by the NIS-Elements Basic Research acquisition and analysis software package (Nikon Instruments, Melville, NY).

For immunoperoxidase staining, permeabilized sections were first treated with 1% hydrogen peroxide in methanol to block endogenous tissue peroxidase, and then blocked in 2% NGS. Slides were washed, incubated with anti-GFAP (1:250), anti-synaptophysin (1:150) or anti-MAP2 (1:100) for 1 h at room temperature, washed again, and then treated with biotin-labeled goat anti-mouse IgG (Vector Laboratories, Burlingame, CA) at a 1:200 dilution for another hour at room temperature. These steps were followed by sequential incubations with avidin-DH-biotin complex (Vector Laboratories) and then 0.5 mg/ml 3,3′-diaminobenzidine (Sigma-Aldrich, St. Louis, MO) in PBS containing 0.01% hydrogen peroxide. All slides were counterstained with hematoxylin and mounted with coverslips for light microscopy. Selected slides were imaged using a Nikon Ti-U inverted microscope equipped with a Nikon DS-Fi-1 digital camera and supported by the NIS-Elements Basic Research acquisition and analysis software package (Nikon Instruments Inc., Melville, NY).

Spinal cord samples for EM were post-fixed overnight in 2.5% glutaraldehyde in 0.1 M Sorenson′s buffer, pH 7.4, at 4°C, and then incubated in 1% osmium tetroxide. After numerous washes in double distilled water, samples were stained with 3% uranyl acetate for 1 h, dehydrated in increasing concentrations of ethanol, rinsed twice in propylene oxide, and embedded in epoxy resin for sectioning. Seventy nanometer sections were stained with uranyl acetate and lead citrate and examined with a Philips CM100 transmission electron microscope at 60 kV. Images were recorded using an ORCA-HR digital camera (Hamamatsu Photonics, Bridgewater, NJ) operated using AMT software (Advanced Microscopy Techniques Corp., Danvers, MA). Motor neurons were identified in lumbar ventral gray matter based on their characteristic morphology, and cell outlines measured by quantitative morphometry. Synaptic density per 100 μm of motor neuron cell contour was counted in 10 cells per animal and 3 animals per experimental group. Each axosomatic synapse was defined as having both a post-synaptic density and multiple presynaptic vesicles. Synapses that had retracted from an adjacent cell body were identified based on the presence of synaptic vesicles only.

### RNA isolation and quantitative real-time PCR

RNA was extracted from the spinal cords of 5 naïve mice and 5 animals at each stage of EAE using TRIzol reagent (Invitrogen, Carlsbad, CA) and precipitated with isopropyl alcohol. After washing with 75% ethanol, RNA was suspended in RNase-free water and a cDNA template was synthesized from 2 μg of each sample using the SuperScript III First-Strand Synthesis System for RT-PCR (Invitrogen). Quantitative real-time PCR was then performed from each cDNA (20 μl reaction/well) using Taqman master mix and a Bio-Rad iQ5 cycler (Hercules, CA). Real-time PCR probes and primer sequences were designed to avoid amplification of any contaminating genomic DNA and were obtained from Integrated DNA Technologies (Coralville, IA) with the following sequences:

*sparcl1* probe:5′-AGGCTGAAAAACCTTCAGCA-3′,
*sparcl1* sense: 5′-CTGACCACTCCAACCCAACT-3′,
*sparcl1* antisense: 5′-TCCTCATCCTTCAGGTCCAC-3′
*sparc* probe: 5′-AAACATGGCAAGGTGTGTGA-3′
*sparc* sense: 5′-AATTTGAGGACGGTGCAGAG-3′
*sparc* antisense: 5′-AAGTGGCAGGAAGAGTCGAA-3′
β*-actin* probe: 5′-CCAGCCAGGTCCAGACGCAG-3′
β*-actin* sense: 5′-TGTGCTGTCCCTGTATGC-3′
β*-actin* antisense: 5′-ATGAGGTAGTCTGTCAGGTC-3′

All probes contained 5′ 6-FAM and 3′ Black Hole Quencher modifications. Levels of *sparcl1* and *sparc* transcripts were calculated relative to β*-actin* using the following formula: 2^[Ct (β-actin)–Ct (Target gene)] × 1000, where Ct is the threshold cycle at which the fluorescent signal became significantly higher than background. Results presented reflect either relative tissue *sparcl1* or *sparc* mRNA expression or the *sparcl1/sparc* transcript expression ratio in a minimum of 4 samples per group.

### Primary astrocyte cultures

Primary astrocytes were cultured from the cortices of 2–6 days old C57BL/6 mice as previously described (Rainey-Barger et al., 2011). Briefly, cortices were minced and trypsinized into a single cell suspension and cells were plated into T-150 tissue culture flasks in DMEM supplemented with 10% fetal bovine serum (FBS), 2 mM L-glutamine, OPI media supplement [1 mM oxaloacetate, 0.45 mM pyruvate, 0.2 U/ml insulin (Sigma-Aldrich)] and antibiotics. After reaching ~70% confluency, flasks were shaken overnight at 200 RPM at 37°C to remove microglia from the more firmly adherent astrocytes. The attached cells were then trypsinized and cultured in medium supplemented with 0.1 mM L-leucine methyl ester (L-LME) to eliminate remaining microglia. After 3 passages, cells were harvested, counted and cultured overnight in 24-well plates (3 × 10^5^ cells/well). The next day, medium was changed and cells were mock treated or stimulated with recombinant murine cytokines (R&D Systems) at varying concentrations. Culture supernatants were collected at defined intervals and stored at −80°C until further use.

### Measurement of SPARC and SPARCL1 concentrations in tissue lysates and astrocyte culture supernatants

Spinal cord homogenates were prepared as described for Western blots and used for ELISA assays. Astrocyte culture supernatants were also analyzed directly by ELISA. Tissue-derived or astrocyte-secreted SPARCL1 or SPARC levels were quantified using commercial ELISA kits (Antibodies-Online, Inc., Atlanta, GA) according to the manufacturer's instructions. The lower limit of detection for these assays was 78 pg/ml or pg/mg tissue protein. All tissue results reflect SPARCL1:SPARC concentration ratios in a minimum of 4 samples per group. Results in culture supernatants reflect either absolute SPARCL1 or SPARC concentrations, fold concentration changes in cytokine-treated cells compared to untreated cells, or SPARCL1:SPARC concentration ratios. All culture supernatant data shown represent a single experiment with a minimum of 4 experimental replicates per condition performed a minimum of 2 times.

### Measurement of tissue cytokine and chemokine concentrations by luminex assays

Spinal cord homogenates were prepared as described for Western blots and ELISA assays, and used for subsequent Luminex analysis. The Milliplex mouse 32-plex cytokine detection system (EMD Millipore, Billerica, Massachusetts, USA) was used according to the manufacturer's instructions to quantify cytokine and chemokine levels directly in spinal tissue extracts. Plates were read on a Luminex 200 instrument (EMD Millipore), and cytokine and chemokine concentrations (pg/ml) calculated by the BioPlex manager software (BioRad) using standard curves. Results presented reflect the mean ± SEM of cytokine or chemokine quantities per milligram of total protein extracted from spinal cord tissue from 3 animals at each time point. The lower limit of detection for these assays was 4.5 pg/ml.

### Statistical analyses

The Prism 5.0 software package (GraphPad Software, La Jolla, CA) was used for all statistical analyses. Results are expressed as mean ± SEM. A one-way analysis of variance (ANOVA) with a *post-hoc* Bonferroni's multiple comparison test was used to assess for differences over time. A Two-way ANOVA was used to assess for differences between groups over time. In all cases, differences at a *p* < 0.05 level were considered significant.

## Results

### Mice with relapsing EAE exhibit dynamic astrocytic and synaptic changes in lumbar spinal gray matter

**Figure 1:**
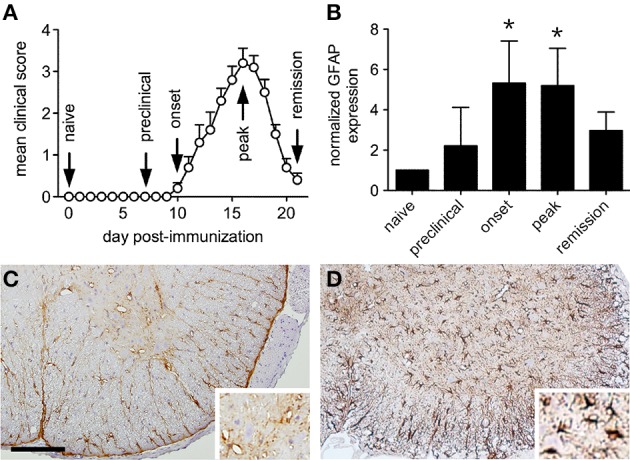
**Astrocytes are activated in both gray and white matter of the lumbar ventral spinal cord during relapsing EAE**. **(A)** The clinical course of relapsing EAE induced by active PLP peptide immunization of SJL mice shows near complete resolution of paralysis over only a few days (*n* = 20). **(B)** Normalized spinal cord GFAP levels at defined stages of relapsing EAE shows evidence of astrocyte activation at disease onset (*n* = 5 samples per disease stage). **(C)** Representative immunohistochemical staining for GFAP expression in naïve SJL spinal cord shows expression in quiescent-appearing cells that predominate in white matter. Insert shows modest signal in ventral gray matter. **(D)** Representative GFAP staining of SJL spinal cord at peak EAE shows increased signal in both white and gray matter. Insert shows numerous activated GFAP+ astrocytes in ventral gray matter. ^*^p < 0.05 compared to preclinical levels, Bar = 100 μm.

Immunization of female SJL mice with the myelin peptide, PLP_139−151_, produced a relapsing form of EAE where the initial hind limb paralysis remitted from peak disease severity over just a few days (Figure 1A). In this disease setting, we found that astrocyte activation in the lumbar spinal cord, as assessed by Western blot expression of the intermediate filament protein, GFAP, preceded the onset of symptoms, reached a peak as hind limb paralysis emerged, and declined with clinical remission (Figure 1B). While inflammatory cell infiltration predominated in the surrounding white matter tracts of mice with relapsing EAE (data not shown), GFAP expression changes were identified by immunohistochemistry in both gray and white matter regions of the lumbar spinal cord at peak disease (Figure 1D) compared to naïve controls (Figure 1C). These dynamic astroglial responses in spinal gray matter raised the possibility that interactions with adjacent neurons might also change over the course of relapse and remission.

**Figure 2:**
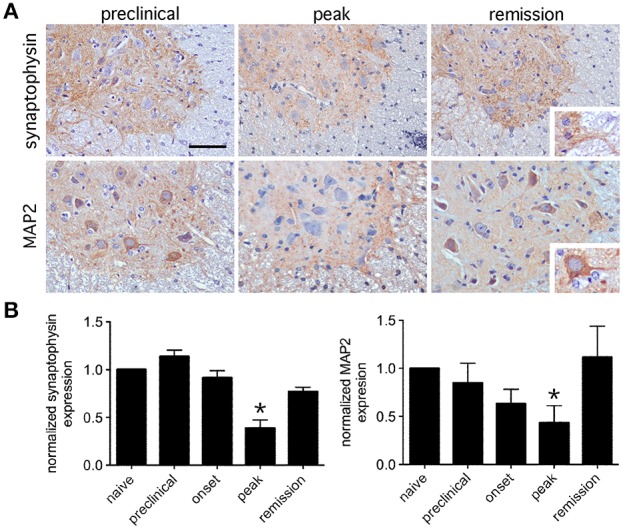
**Expression of synaptic proteins in lumbar spinal gray matter is dynamically regulated during relapsing EAE**. **(A)** Representative immunohistochemical staining for the presynaptic protein, synaptophysin, and the postsynaptic protein, MAP2, shows that both proteins localize to lumbar spinal gray matter at varying stages of relapsing EAE in SJL mice. Inserts show that synaptophysin labels gray matter neuropil while MAP2 labels neuronal cell bodies and proximal dendrites. **(B)** Normalized expression of both proteins shows reduced levels at peak disease, but notable recovery by the time of disease remission only a few days later (*n* = 5 samples per disease stage). Bar = 80 μm, ^*^p < 0.05 compared to preclinical levels.

**Figure 3:**
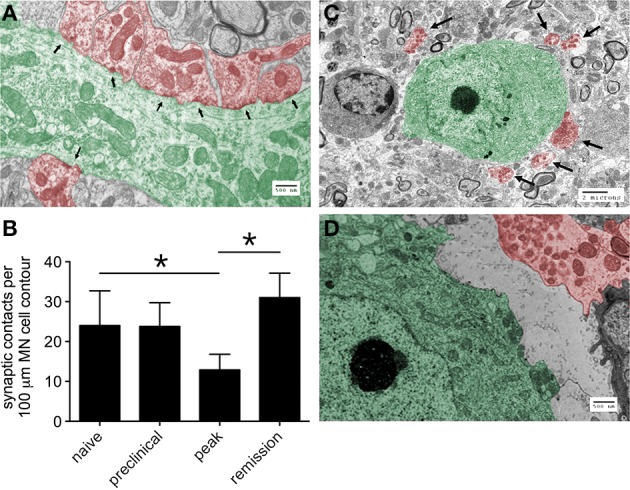
**The number of synapses in direct contact with the proximal dendrites or cell bodies of lumbar ventral motor neurons changes over the course of relapsing EAE**. **(A)** A representative electron micrographic image showing the proximal dendrite of a ventral motor neuron (green) with multiple postsynaptic densities (arrows) indicative of individual synapses (red), Bar = 500 nm. **(B)** Quantification of these synaptic contacts at different stages of relapsing EAE shows evidence of synaptic retraction from the perikarya of motor neurons (MN) at peak disease with full reestablishment of these structures around MN several days later in the setting of disease remission (*n* = 10 MN from each of 3 mice at each disease stage), ^*^p < 0.05 naïve versus peak and peak versus remission. **(C)** A representative electron micrograph at peak disease shows many synapses (red, with arrows) displaced from a motor neuron cell body (green), Bar = 2 μm. **(D)** Another representative electron micrograph at higher magnification shows the physical separation of an axosomatic synapse (red) from the cell body of a ventral motor neuron (green), without any other cellular process interposed between the two structures, Bar = 500 nm.

To survey the general integrity of synapses in the lumbar spinal cords of mice with relapsing EAE, tissue expression of the pre-synaptic neuronal protein, synaptophysin, and the post-synaptic neuronal protein, MAP2, were assessed. By immunohistochemistry both proteins localized exclusively to spinal gray matter (Figure 2A), and by Western blot both proteins were dynamically regulated in directions opposite to paralysis severity (i.e., levels declined as paralysis scores increased, and vice versa) (Figure 2B). Focusing on the motor pathway at an ultrastructural level, the actual number of synapses found in direct contact with the perikarya of lumbar spinal motor neurons were notably reduced at peak disease but recovered as motor function returned back to normal (Figures 3A,B). At peak disease, few of these axosomatic synapses showed evidence that another cell had interposed a process between itself and the neuronal cell body; most appeared to have simply retracted from the cell perimeter (Figures 3C,D). Together, these data suggest that physical changes to the synapses projecting directly onto ventral motor neurons or their dendritic processes could contribute to the fluctuating paralysis that typifies relapsing-remitting EAE. They also show that dynamic modulation of these synaptic connections occurs in both temporal and spatial proximity to reactive astrocytes in surrounding gray matter.

### Altered expression of SPARCL1 and SPARC mRNA and protein levels in the lumbar spinal cord over the course of EAE

**Figure 4:**
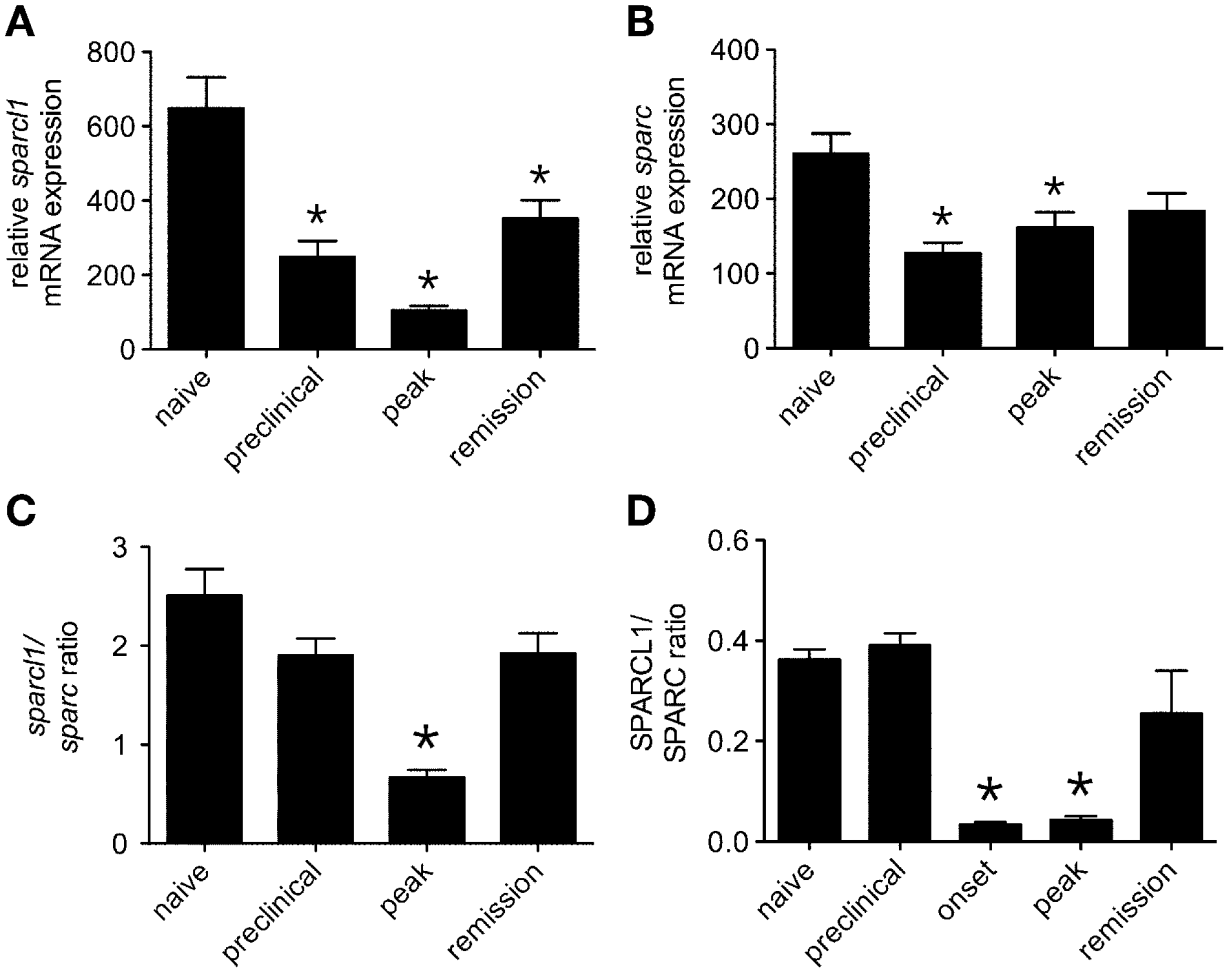
**Expression of both ***sparcl1*** and ***sparc*** transcripts and SPARCL1 and SPARC proteins changes in lumbar spinal cord tissue over the course of relapsing EAE in SJL mice**. **(A)** Relative *sparcl1* mRNA expression fluxes over the course of relapsing EAE (*n* = 5 mice per disease stage). **(B)** Relative *sparc* mRNA expression also changes over the course of relapsing EAE (*n* = 5 mice per disease stage). **(C)** The calculated *sparcl1* to *sparc* mRNA expression ratio shifts in favor of synapse inhibition at peak disease. **(D)** The same change in the SPARCL1 to SPARC protein concentration ratios is seen over the course of relapsing EAE (*n* = 5 mice per disease stage). ^*^p < 0.05 compared to levels found in naïve spinal cord.

The matricellular proteins, SPARC and SPARCL1, are produced by astrocytes and regulate excitatory synaptogenesis *in vitro* and in the developing CNS *in vivo* (Kucukdereli et al., 2011). While SPARCL1 directly promotes excitatory synapse formation, SPARC specifically antagonizes its synaptogenic activity (Kucukdereli et al., 2011). Both proteins are expressed in the adult CNS (Lloyd-Burton and Roskams, 2012), although their direct effects over existing excitatory synapses has not been reported. Given dynamic changes to both astrocytes and synapses in the lumbar gray matter of mice with relapsing EAE (Figures 1–3), we investigated whether local tissue expression of either SPARCL1 or SPARC changed over the course of this disease. Real-time PCR analysis showed that *sparcl1* transcript levels declined in lumbar spinal cord with the onset of CNS inflammation, were further suppressed at peak disease, but then increased during remission in SJL mice with EAE (Figure 4A). Transcript levels of its known inhibitor, *sparc*, also declined during the preclinical stage of disease but increased thereafter (Figure 4B). When *sparcl1:sparc* transcript ratios were calculated, a dramatic, but transient, shift favoring synapse inhibition was seen at peak disease (Figure 4C). A similar pattern in the SPARCL1:SPARC protein expression ratios was observed in spinal cord tissues over the course of disease when each protein was measured by ELISA (Figure 4D).

**Figure 5:**
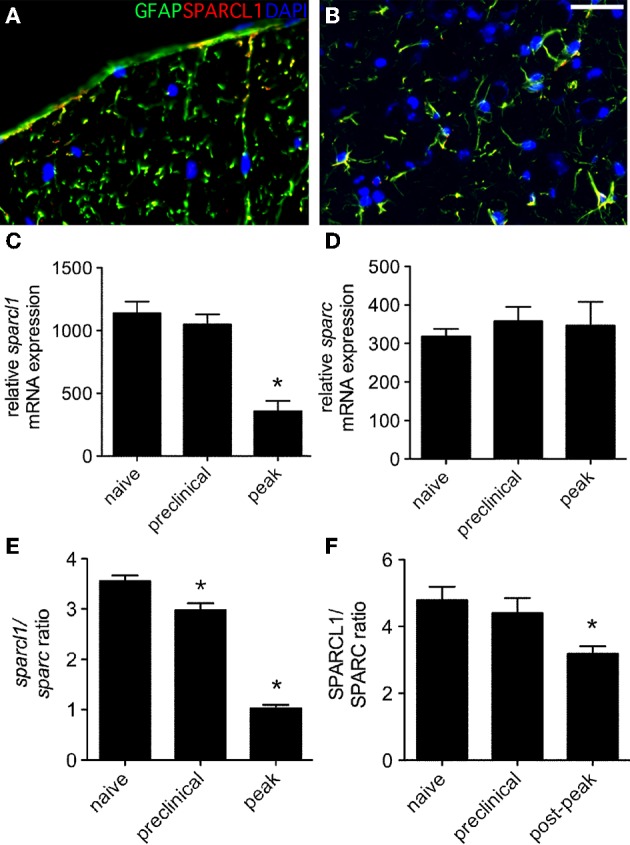
**Expression of ***sparcl1*** and ***sparc*** transcripts and SPARCL1 and SPARC proteins changes in lumbar spinal cord tissue over the course of non-relapsing EAE in C57BL/6 mice**. **(A,B)** Representative immunofluorescence shows SPARCL1 expression co-localizes with GFAP staining in both white and gray matter of the spinal cord, Bar = 40 μm. **(C)** Relative *sparcl1* mRNA expression fluxes over the course of non-relapsing EAE (*n* = 5 mice per disease stage). **(D)** Relative *sparc* mRNA expression does not change over the course of non-relapsing EAE (*n* = 5 mice per disease stage). **(E)** The calculated *sparcl1* to *sparc* mRNA expression ratio shifts in favor of synapse inhibition at peak disease. **(F)** A similar change in the SPARCL1 to SPARC protein concentration ratios is seen over the course of non-relapsing EAE (*n* = 5 mice per disease stage). ^*^p < 0.05 compared to levels found in naïve spinal cord.

When non-relapsing EAE was induced in C57BL/6 mice following immunization with the MOG_35−55_ peptide, SPARCL1 co-localized with GFAP expression in both gray and white matter (Figures 5A,B), and *sparcl1* transcripts also declined in the lumbar spinal cord as mice transitioned from preclinical to peak disease (Figure 5C). In this setting, *sparc* levels did not change over time (Figure 5D), but the *sparcl1:sparc* transcript ratio and the SPARCL1:SPARC protein ratio also declined as disease symptoms became severe (Figures 5E,F). Together, these data show that factors present in the spinal cord suppress astrocyte production of synaptogenic proteins during peak EAE even as these cells assume an activated phenotype.

### Pro- and anti-inflammatory cytokines and chemokines get induced in the spinal cord during EAE, and T cell-derived factors regulate astrocyte production of SPARCL1 and SPARC *in vitro*

**Figure 6:**
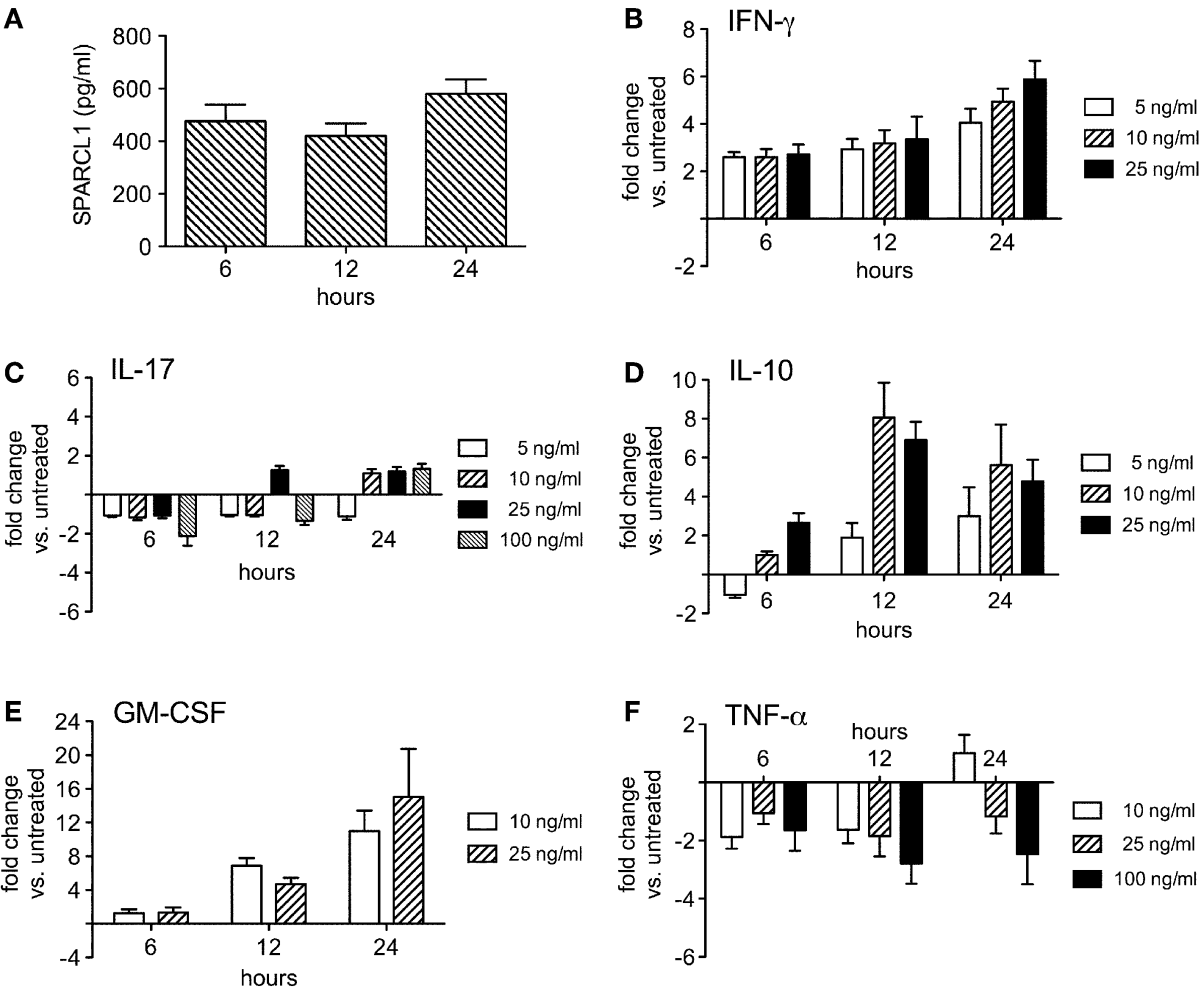
**T cell-derived cytokines regulate astrocyte production of SPARCL1 in a complex manner ***in vitro*****. **(A)** Astrocytes spontaneously secrete measurable amounts of SPARCL1 into culture supernatants (*n* = 4 experimental replicates per time point). **(B)** Interferon-gamma (IFN-γ) modestly increases astrocyte SPARCL1 release (*n* = 3 experimental replicates per time point), *p* = 0.003 comparing changes over time. **(C)** Interleukin (IL)-17 has no significant effect on astrocyte SPARCL1 production (*n* = 3 experimental replicates per time point). **(D)** IL-10 potently induces astrocyte SPARCL1 production (*n* = 3 experimental replicates per time point), *p* = 0.0003 comparing changes over time, *p* = 0.002 comparing fold change differences. **(E)** Granulocyte macrophage colony stimulating factor (GM-CSF) potently induces astrocyte SPARCL1 production (*n* = 3 experimental replicates per time point), *p* = 0.0024 comparing changes over time. **(F)** Tumor necrosis factor (TNF)-α suppresses astrocyte SPARCL1 production (*n* = 3 experimental replicates per time point), *p* = 0.0384 comparing fold change differences.

Hind limb paralysis in mice with EAE is driven by local CNS inflammation initiated by myelin-specific CD4+ T cells. In this setting, a wide range of cytokines and chemokines get induced in the spinal cord at peak disease (Table 1). We used primary glial cell cultures to investigate whether some of these mediators directly altered astrocyte production of SPARCL1 or SPARC, focusing on those factors made directly by encephalitogenic CD4+ T cells. Primary astrocytes released substantial amounts of SPARCL1 into culture supernatants without any direct provocation (Figure 6A). When cells were treated with varying concentrations of T cell-derived cytokines, a wide range of effects on SPARCL1 production was observed. IFN-γ, the prototype Th1 pro-inflammatory cytokine, modestly increased SPARCL1 levels in astrocyte culture supernatant (Figure 6B), while the canonical Th17 cytokine, IL-17, had no effects on SPARCL1 release (Figure 6C). The anti-inflammatory cytokine, IL-10, potently increased astrocyte SPARCL1 production (Figure 6D). Granulocyte-macrophage colony stimulating factor (GM-CSF) also potently increased SPARCL1 levels (Figure 6E), even though it also serves as an important effector in Th17-driven EAE by mobilizing myeloid cells to the CNS (Kroenke et al., 2008; Codarri et al., 2011). Finally, TNF-α suppressed SPARCL1 release (Figure 6F). We conclude that as a group, T cell-derived cytokines exert complex effects on astrocyte SPARCL1 release. Practical considerations prevented us from examining the extent to which the many candidate non-T cell-derived cytokines and chemokines influenced SPARCL1 production. Nonetheless, astrocytes express a wide range of cytokine and chemokine receptors meaning that other mediators could also contribute to the effects observed *in vivo* (Farina et al., 2007).

**Table 1:** **Concentrations of various inflammatory mediators in the spinal cords of mice at peak EAE (***n*** = 3)**.

**Mediator**	**Concentration (pg/mg of total spinal cord tissue protein (±SEM)**
IFN-γ	36.39 ± 18.69
IL-1α	144.41 ± 80.26
IL-1β	6.20 ± 6.20
IL-2	77.41 ± 28.79
IL-6	5.47 ± 3.81
IL-7	6.80 ± 3.71
IL-9	2020.41 ± 938.06
IL-10	284.42 ± 262.74
IL-12p40	10.73 ± 6.25
IL-12p70	30.29 ± 16.92
IL-13	144.62 ± 94.11
IL-15	44.38 ± 22.21
IL-17	70.03 ± 50.18
CCL2	66.06 ± 28.42
CCL3	43.99 ± 27.44
CCL4	18.07 ± 9.61
CCL5	25.97 ± 12.92
CCL11	26.22 ± 13.32
CXCL1	101.45 ± 28.88
CXCL2	59.31 ± 38.81
CXCL5	25.01 ± 25.01
CXCL9	883.77 ± 529.62
CXCL10	260.23 ± 151.37
GM-CSF	43.91 ± 33.54
M-CSF	41.25 ± 25.74
TNF-α	94.31 ± 50.79
VEGF	8.36 ± 6.60

*Below the stated assay limit of detection: G-CSF, IL-3, IL-4, IL-5, LIF*.

**Figure 7:**
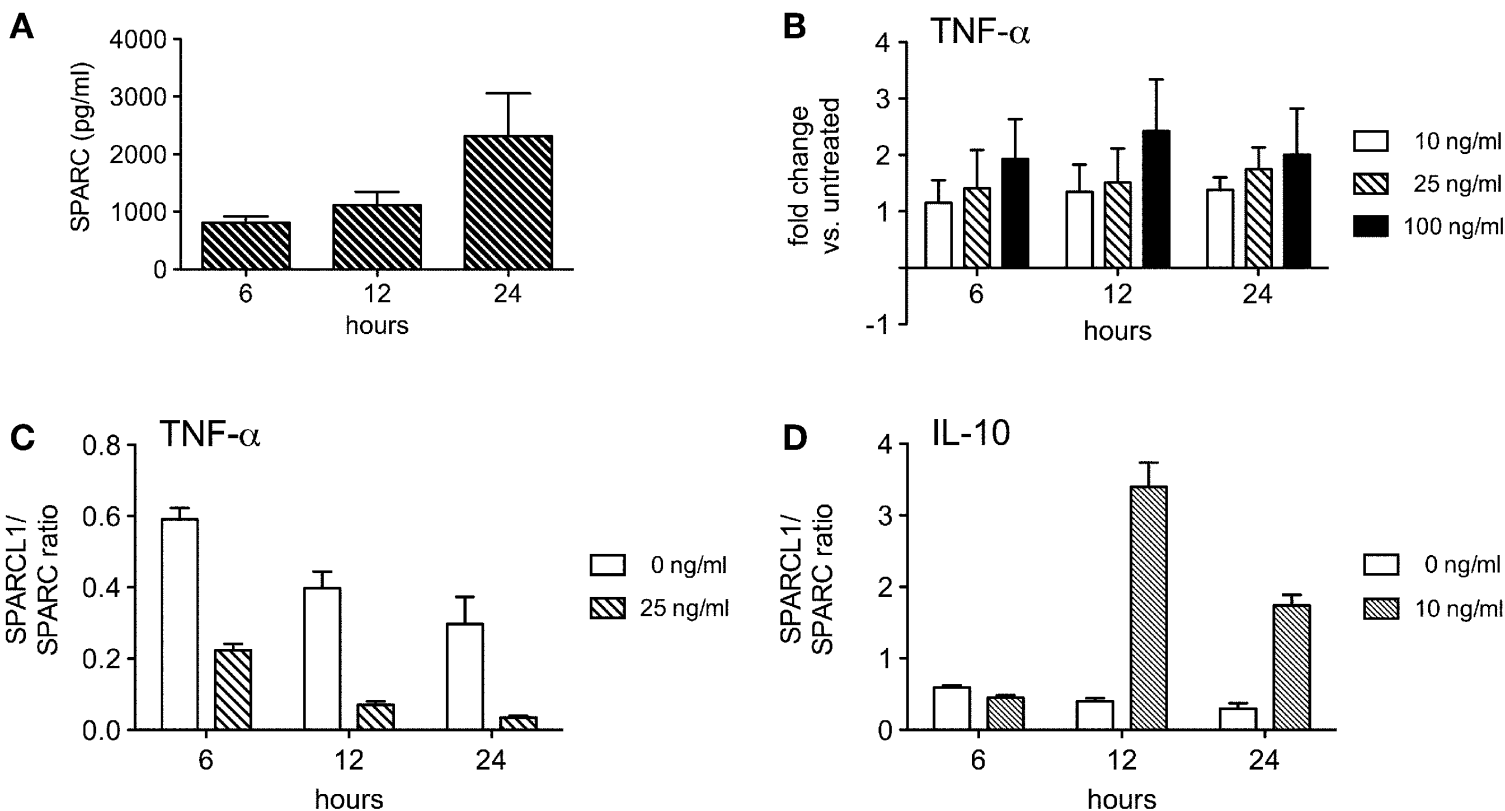
**Some T cell-derived cytokines regulate astrocyte production of SPARC ***in vitro*****. **(A)** Astrocytes spontaneously secrete measurable amounts of SPARC into culture supernatants (*n* = 4 experimental replicates per time point). **(B)** TNF-α modestly induces astrocyte SPARC production (*n* = 3 experimental replicates per time point). **(C)** TNF-α suppresses the ratio of SPARCL1 to SPARC made by astrocytes over time, *p* < 0.0001 comparing ratio differences, *p* = 0.0002 comparing changes over time. **(D)** IL-10 augments the ratio of SPARCL1 to SPARC made by astrocytes over time, *p* < 0.0001 comparing ratio differences, *p* < 0.0001 comparing changes over time.

Spontaneous astrocyte production of SPARC was also robust (Figure 7A), although fewer of the cytokines tested modulated its release. TNF-α modestly increased SPARC production by these cells (Figure 7B), thereby shifting the SPARCL1:SPARC concentration ratio more in favor of synapse inhibition (Figure 7C). An opposite effect on this ratio was induced by IL-10 (Figure 7D), a change largely driven by its induction of SPARCL1 in astrocytes (Figure 6D). Both IFN-γ and IL-17 had no significant effect on SPARC release (data not shown). Nonetheless, these data show that *in vitro* production of this anti-synaptogenic molecule by astrocytes is also under some dynamic control by T cell-derived inflammatory mediators.

## Discussion

Gray matter pathology is increasingly recognized in MS patients, even if its origins remain poorly understood. In some circumstances, axonal damage in white matter lesions leads to retrograde degeneration in the cerebral cortex, eventually causing loss of cortical volume (Calabrese et al., 2015). In other settings, gray matter damage occurs fully independent of white matter injury causing what has been described as “outside-in” brain pathology (Calabrese et al., 2015). Proposed mechanisms for this latter form of damage include: (a) immune-mediated events arising from perivascular infiltrates directly in gray matter parenchyma or in focal lymphoid-like structures found in the overlying meninges (Serafini et al., 2004; Magliozzi et al., 2010; Lucchinetti et al., 2011; Calabrese et al., 2015), (b) aberrant activation of gray matter microglia (Peterson et al., 2001; Dutta and Trapp, 2007), and/or (c) activation of non-inflammatory degenerative pathways (some possibly related to mitochondrial dysfunction) targeting neurons, oligodendrocytes or astrocytes that only trigger inflammation as a secondary event (Barnett and Prineas, 2004; Mahad et al., 2008; Henderson et al., 2009). In all such scenarios, primary neuronal loss in cortical gray matter would cause anterograde degeneration of axons in downstream white matter pathways. Imaging data obtained in early RRMS patients with comparable white matter lesion loads show that clinical disability progresses faster in those individuals with more widespread gray matter atrophy (Calabrese et al., 2015). These and other findings argue that the mechanisms underlying gray matter pathology must be understood and accounted for by new therapies designed to slow or halt MS progression.

Synapses are the main interface whereby networks of neurons interact with each other during normal nervous system function. More and more studies find synaptic abnormalities and gray matter lesions in MS patients (Vercellino, 2005; Stadelmann et al., 2008; Michailidou et al., 2015). Dysfunction or loss of synapses could allow for the emergence of neurological deficits without corresponding white matter pathology, and dynamic changes to synapses could easily produce deficits that fluctuate over time. While direct imaging synaptic changes in living humans is not yet feasible, two-photon intravital microscopy has been used to visualize axonal boutons and dendritic spines in the cortices of mice with EAE in real time. One such study showed notable synaptic instability during disease, often starting well before immune cell infiltration or the onset of overt symptoms (Yang et al., 2013). TNF-α was implicated in these cortical synaptic changes as the fluctuations were suppressed with a TNF inhibitor (Yang et al., 2013). Even in the absence of a disease such as EAE, TNF-α has been shown to regulate homeostatic synaptic scaling in a way that optimizes the performance of neural networks (Stellwagen and Malenka, 2006). Thus, at least one inflammatory mediator is closely linked to the structure and function of synapses during both health and disease.

A recent study by Chen et al. demonstrated that activated microglia transiently displace synapses from the perikarya of cortical neurons as a neuroprotective response (Chen et al., 2014). In this setting, microglia closely oppose themselves to neuronal cell bodies and physically displace inhibitory GABAergic synapses, thereby increasing the synchronized firing of cortical neurons and augmenting their expression of antiapoptotic and neurotrophic molecules (Chen et al., 2014). This process differs from the pruning of synapses by microglia during development in that it rapidly resolves itself and does not require complement deposition (Chen et al., 2014). Although we find activated microglia in lumbar gray matter during EAE (data not shown), we do not find convincing ultrastructural evidence that microglial cell processes interpose themselves between motor neuron cell bodies and displaced presynaptic terminals (Figures 3C,D). We believe our data are more compatible with a transient disruption of a preexisting synaptic maintenance system, rather than any active synaptic stripping by another cell type.

Astrocytes are now recognized to support the formation, maturation, activity, and elimination of neuronal synapses (Clarke and Barres, 2013; Haydon and Nedergaard, 2014; De Pittà et al., 2015). While such cellular interactions are most apparent during development (Allen and Barres, 2005; Christopherson et al., 2005; Eroglu et al., 2009; Kucukdereli et al., 2011; Allen et al., 2012; Clarke and Barres, 2013; Haydon and Nedergaard, 2014; De Pittà et al., 2015), there is growing evidence they continue into adulthood. For example, astrocytes were recently shown to continuously engulf synapses in the normal adult brain (Chung et al., 2013). This active synaptic remodeling implies that astrocytes could play an important role in basic brain functions such as learning and memory. Furthermore, astrocyte-driven synaptic plasticity appears important for the recovery from acute CNS injury; one study showed that the astrocyte proteins, thrombospondin (TSP)-1 and TSP-2, facilitate the reestablishment of synapses and promote the functional recovery of mice following experimental stroke (Liauw et al., 2008). Our data show a dynamic regulation of astrocyte-derived SPARCL1 and SPARC by local factors in the spinal cord, implying that dysfunction of these cellular processes is intrinsic to EAE itself. In other words, while astrocyte-driven synaptic plasticity may be important for both normal development and the host response to acute CNS injury, this adaptive process can also be subverted as part of a disease process. An improved understanding of when and how to shift astrocytes toward the maintenance of synapses and away from synapse inhibition could open up novel therapeutic strategies for disorders such as RRMS.

Our study correlates *in vitro* and *in vivo* findings to draw links between changing levels of astrocyte-derived proteins involved in synaptic homeostasis, local tissue inflammation and EAE disease severity. It does do not confirm any cause-and-effect link between these events. Nonetheless, the data do support our hypothesis that local tissue inflammation influences both synaptic plasticity in lumbar ventral gray matter as well as clinical symptoms in mice with EAE by dynamically regulating astrocyte production of proteins such as SPARCL1 and SPARC. Our *in vitro* data show that T cell-derived cytokines can directly control astrocyte production of these matricellular proteins, and it seems likely that tissue expression of these proteins over the course of EAE is the result of local astrocytes integrating the effects of the inflammatory milieu around them. Our ongoing studies aim to show that inducible over-expression or direct replacement of SPARCL1, or timed neutralization of SPARC, can ameliorate the severity of EAE paralysis in order to more directly prove these astrocytic changes contribute to disease. If confirmed, this would represent the first therapeutic strategy that direct targets glial cells in this disorder.

## Author contributions

PB, SH, LC, and DI all contributed to the design, acquisition and/or interpretation of data for the study; SH and DI contributed to drafting or revising the contents of this report; PB, SH, LC, and DI all approved the final version of this paper; PB, SH, LC and DI all agree to be accountable for all aspects of this work.

### Conflict of interest statement

The authors declare that the research was conducted in the absence of any commercial or financial relationships that could be construed as a potential conflict of interest.
